# Population Pharmacokinetics and Pharmacodynamics of Depemokimab in People with Asthma and Chronic Rhinosinusitis with Nasal Polyps

**DOI:** 10.1002/jcph.70187

**Published:** 2026-07-30

**Authors:** Anders Thorsted, Lénaïg Tanneau, Alexandra Lavalley‐Morelle, Richard Follows, Loretta Jacques, Nicholas Bird, Philippe Gevaert, Ian Pavord, Jakob Ribbing, Anubha Gupta, Peter Howarth, Stein Schalkwijk

**Affiliations:** ^1^ Clinical Pharmacology Modelling and Simulation GSK Solna Sweden; ^2^ Pharmetheus AB Uppsala Sweden; ^3^ Clinical Research Respiratory, Inflammation and Immunology Research Unit (RIIRU) GSK London UK; ^4^ Biostatistics GSK London UK; ^5^ Upper Airways Research Laboratory Department of Head and Skin Ghent University Ghent Belgium; ^6^ Oxford Respiratory NIHR Biomedical Research Centre Nuffield Department of Clinical Medicine University of Oxford Oxford UK; ^7^ Clinical Pharmacology Modelling and Simulation GSK Stevenage UK; ^8^ Global Medical Affairs Specialty Care GSK London UK; ^9^ Clinical Pharmacology Modelling and Simulation GSK Amsterdam Netherlands

**Keywords:** biologicals, modeling and simulation, NONMEM, population analysis, pharmacokinetic‐pharmacodynamic

## Abstract

Depemokimab is the first ultra‐long‐acting biologic with enhanced interleukin‐5 (IL‐5) binding affinity, high potency, and extended half‐life, enabling twice‐yearly dosing. Depemokimab has demonstrated efficacy and safety in patients with asthma and chronic rhinosinusitis with nasal polyps. These analyses aimed to characterize the pharmacokinetic/pharmacodynamic (PK/PD) profile of depemokimab. Data from three Phase I studies (NCT03287310/NCT05140200/NCT05602025) and four Phase III studies (SWIFT‐1/‐2: NCT04719832/NCT04718103; ANCHOR‐1/‐2: NCT05274750/NCT05281523) were used to develop a PK model for depemokimab and PK/PD model for blood eosinophil count (BEC) reduction, a key biomarker of type 2 inflammation. Participants received subcutaneous depemokimab or placebo (one dose in Phase I [2‐300 mg in NCT03287310; 100 mg or 300 mg in NCT05140200/NCT05602025] and two 100 mg doses in Phase III) and ≥1 post‐first‐dose PK/PD (BEC) observation. PK analysis included 961 participants and PK/PD analysis included 1324 participants. Predicted geometric mean half‐life was 47 days, with negligible accumulation in PK. Depemokimab 100 mg rapidly reduced BEC to <25% of baseline, with a small increase observed at the end of the 26‐week dosing interval. No clinically relevant covariates were identified for either model. All participants had trough depemokimab concentrations above the half maximal effective concentration (EC_50_) at the end of the dosing interval (90% above the EC_90_ level). PK/PD modeling showed that single‐dose depemokimab 100 mg provides durable BEC reduction throughout a 26‐week dosing interval, suggesting sustained suppression of IL‐5 activity. These data support twice‐yearly recommended dosing for depemokimab 100 mg, with no dose adjustments required based on intrinsic or extrinsic factors.

## Introduction

Interleukin‐5 (IL‐5) is a core cytokine in type 2 inflammation and has broad multidirectional effects (direct and indirect) on eosinophils, epithelial cells, mast cells, plasma cells, basophils, ILC2s, regulatory T cells, smooth muscle cells, neutrophils, and fibroblasts.^1^ As such, IL‐5 plays a key role in the pathogenesis of airway diseases driven by underlying type 2 inflammation, such as severe asthma and chronic rhinosinusitis with nasal polyps (CRSwNP).^1^, ^2^ Blood eosinophil count provides an indication of IL‐5 activity and as a result is a key biomarker for type 2 inflammation in these diseases.^3^

Depemokimab is the first ultra‐long‐acting biologic with enhanced IL‐5 binding affinity, high potency, and extended half‐life, enabling twice‐yearly dosing and sustained inhibition of IL‐5 activity (as indicated by reduction in blood eosinophil count) in people with type 2‐driven diseases.^4^, ^5^ This dosing approach is aimed at reducing treatment burden (compared with the bi‐weekly/monthly administration typical of most monoclonal antibody therapies) and potentially improving adherence and persistence to biologic therapies, which could translate into long‐term real‐world benefits.^6^ Depemokimab binds to the same epitope as the established anti‐IL‐5 monoclonal antibody, mepolizumab, which is approved as add‐on maintenance treatment for type 2 asthma characterized by blood eosinophils, and for CRSwNP.^7^, ^8^ Depemokimab includes three particular amino‐acids (Gly31, Ser32 and Gly99) in the heavy chain complementarity‐determining regions which were anticipated to increase IL‐5‐binding affinity, and also a clinically‐validated YTE (Tyr252/Thr254/Glu256) in the Fc region to extend the in vivo plasma half‐life.^6^ The YTE‐Fc mutation increases depemokimab's binding to the neonatal Fc receptor (FcRn) in endosomes, enhancing its recycling by FcRn and extending its circulation time in the body.^6^

In the first‐time‐in‐human (FTIH), single‐ascending dose, Phase I study of depemokimab in adult participants with mild‐to‐moderate asthma and elevated blood eosinophils (≥200 cells/µL at screening), depemokimab was shown to be well tolerated with linear and dose proportional pharmacokinetics (PK) at doses of 10‐300 mg.^9^ Results also demonstrated that depemokimab had an extended half‐life of 38‐53 days, and led to a prolonged reduction in blood eosinophil count after a single dose (sustained over 26 weeks with the 100 mg and 300 mg doses).^9^ Depemokimab progressed directly from Phase I to Phase III using a combined model‐informed drug development and quantitative decision‐making approach that combined PK/pharmacodynamic (PD) and clinical trial simulations with the established pharmacology, efficacy, and safety of IL‐5 inhibitors to justify dose selection and trial design.^10^ On the basis of this PK/PD model, a dose of 100 mg administered subcutaneously (SC) every 26 weeks was recommended as a robust dosing regimen for Phase III trials in patients with asthma/CRSwNP.^10^ The Phase III SWIFT‐1/‐2 studies showed that the initial observations translated into clinical benefits in participants with type 2 asthma, with depemokimab (100 mg SC, every 26 weeks) significantly reducing the annualized rate of exacerbations (depemokimab versus placebo pooled rate ratio of 0.46 [95% confidence interval^11^: 0.36 to 0.58]) and reducing blood eosinophil count (82%‐83% reduction versus baseline at Week 52 [blood eosinophil count criteria at baseline: ≥300 cells/µL in past 12 months or ≥150 cells/µL at screening]), which may indicate sustained suppression of IL‐5 activity (a key driver of type 2 inflammation).^5^ Similarly, the Phase III randomized, double‐blind, placebo‐controlled ANCHOR‐1/‐2 studies (which included participants with inadequately controlled CRSwNP) demonstrated that depemokimab (100 mg SC, every 26 weeks) significantly improved clinically relevant endpoints, reducing total nasal polyps score versus placebo by −0.7 (95% CI: −0.9 to −0.4) at Week 52 and nasal obstruction verbal response scale score by −0.24 (95% CI: −0.39 to −0.08) at Weeks 49‐52, supporting its use as a twice‐yearly treatment to reduce CRSwNP disease burden.^4^

PK and PD modeling and simulation studies were performed to characterize the PK/PD relationship between depemokimab plasma concentration and blood eosinophil count (including identification of individual parameters/exposure metrics and the impact of covariates, as well as inter‐individual and residual unexplained variability) and to support the use of the 100 mg dose of depemokimab with twice‐yearly administration. Here we present the results of these analyses, which aimed to develop a population PK model for depemokimab and utilize this to characterize the PK/PD profile of depemokimab based on blood eosinophil count, using data from Phase I and Phase III clinical trials conducted in people with asthma or CRSwNP.

## Methods

### Analysis Design

Data from three Phase I studies and four Phase III studies were included in these analyses. The three Phase I studies included a FTIH randomized, double‐blind, placebo‐controlled, ascending‐dose study of depemokimab (2‐300 mg; single SC doses [liquid in vials]) in European participants with mild‐moderate asthma (GSK ID: 205722; NCT03287310),^9^ an open‐label, parallel‐group study of depemokimab (100 mg or 300 mg; single doses (liquid) administered SC via a prefilled syringe assembled in a safety device, i.e. safety syringe) in healthy volunteers in China (208021; NCT05140200)^12^ and an open‐label, parallel‐group study of depemokimab (100 mg SC, via either safety syringe or autoinjector; single doses [liquid]) in US healthy volunteers (214099; NCT05602025).^13^ These will be referred to hereafter as the FTIH Asthma, China HV, and US HV studies, respectively.

The four Phase III trials were all randomized, double‐blind, placebo‐controlled studies: SWIFT‐1 and SWIFT‐2 investigated the efficacy and safety of depemokimab 100 mg SC (via liquid safety syringe) in participants with type 2 asthma characterized by blood eosinophils (GSK ID: 206713/213744; NCT04719832/NCT04718103)^5^; and ANCHOR‐1 and ANCHOR‐2 investigated the efficacy and safety of depemokimab 100 mg SC (liquid via safety syringe) in participants with inadequately controlled CRSwNP (GSK ID: 217095/218079; NCT05274750/NCT05281523).^4^ These will be referred to hereafter as the SWIFT‐1/‐2 and ANCHOR‐1/‐2 studies, respectively.

### Study Populations

Participants enrolled in the FTIH Asthma study were 18‐65 years of age with a diagnosis of asthma (at least 12 months prior to the study) in sites in the UK or Germany, who had received stable asthma treatment for at least 12 weeks, and who had a pre‐bronchodilator forced expiratory volume in 1 s (FEV_1_) of at least 60% of normal value and a blood eosinophil count of at least 200 cells/µL at screening.^9^ Participants enrolled in the China HV and US HV studies were healthy adult volunteers from China or the US, respectively.^12^, ^13^

Participants enrolled in the global SWIFT‐1/‐2 studies were at least 12 years of age with a diagnosis of asthma, a blood eosinophil count of at least 150 cells/µL at screening or 300 cells/µL during the 12 months prior to the study, airflow obstruction and a history of at least 2 exacerbations in the previous 12 months.^5^ Participants enrolled in the global ANCHOR‐1/‐2 studies were at least 18 years of age with inadequately‐controlled CRSwNP, a bilateral nasal polyps score of ≥5 (≥2 per nasal cavity), severe CRSwNP symptoms (based on nasal obstruction, rhinorrhea, and loss of smell) and previous treatment within the past 2 years (or medical contraindication or intolerance to systemic corticosteroid treatment) or documented prior surgery for CRSwNP.^4^

All trials were conducted in accordance with consensus ethical principles derived from international guidelines including the Declaration of Helsinki and Council for International Organizations of Medical Sciences international ethical guidelines, applicable International Conference on Harmonization and Good Clinical Practice guidelines, and any additional applicable laws and regulations.

Data for participants who received depemokimab from all seven studies were included in the pooled PK analyses, whereas only data from the FTIH Asthma, SWIFT‐1/‐2, and ANCHOR‐1/‐2 studies were included in the PK/PD analyses (i.e. excluding non‐placebo‐controlled studies in healthy volunteers who were not expected to have elevated blood eosinophil counts). The PK analysis population included all participants who received at least one dose of depemokimab and who had at least one post‐first‐dose PK observation. Multiple assessments of PK (between 9 and 16) were made at different timepoints from Day 1 up to 364 days post‐first‐dose depending on the study; a total of 9383 observations were included from 961 participants. For the PK/PD analysis, the blood eosinophil count population included all participants who received at least one dose of depemokimab or placebo, and who had at least one post‐first‐dose blood eosinophil observation. Between 6 and 15 assessments of blood eosinophils were conducted at different timepoints in each study from screening up to 364 days post‐dose; a total of 14,273 observations were included from 1324 participants.

### Bioanalytical Methods

Depemokimab concentrations in human K2EDTA plasma were quantified as described previously.^9^, ^13^ For the FTIH Asthma study, the validated assay had within‐run precision of ≤7.5% and overall accuracy of −7.3% to 3.5% for a detection range of 50‐10,000 ng/mL.^9^ For all other studies, the measured within‐run and between‐run precision of the assay was ≤8.2% and ≤10.4%, respectively. The measured overall accuracy was −3.6% to −8.6%.^13^

### Immunogenicity Assessment

To determine anti‐drug antibody (ADA) positivity, a tiered analyses approach was used for immunogenicity assessment, including a validated binding ADA assay (screening, confirmation, and titration assays) and a validated neutralizing antibody (Nab) assay.^13^ For the FTIH Asthma study, an earlier version of the ADA assay was used which was later found to be susceptible to target interference.^9^

### PK and PK/PD Model Development

For PK model development, data exclusions included pre‐dose samples, those below the LLOQ (0.05 µg/mL for the FTIH Asthma study and 0.1 µg/mL for other studies) and placebo data; outlying samples were also excluded in the PK analysis data set. Observation records with depemokimab PK plasma concentration values below the LLOQ were retained in the derived data file for inclusion in model evaluation using visual predictive checks, with the values set to LLOQ/2. The starting model was a one‐compartment model with first order absorption. A parameter for relative bioavailability with a fixed value of 1 was also included in the model to facilitate the estimation of inter‐individual variability in the extent of absorption, and to allow for study or population differences in the extent of absorption via covariate analysis. Alternative model options were then tested and analyzed for suitability using graphical diagnostics (goodness‐of‐fit plots) and changes in the objective function value to refine and develop the base depemokimab PK model (using a threshold of *p* < 0.05 to include more complex aspects for hierarchical models). Both the structural and statistical models were considered at this stage with regards to inter‐individual variability and residual unexplained variability. Structural and exploratory covariate‐parameter relationships (e.g. for participant characteristics such as age, sex, body weight, race, as well as medication use [e.g. concomitant systemic corticosteroid use], injection site, and study population) were then assessed using a stepwise covariate model building procedure with adaptive scope reduction,^14^ including forward selection and backwards elimination (using *p*‐value thresholds of 0.01 and 0.001, respectively) (further details provided in Supporting Information). Following finalization, the PK model was evaluated including assessment of goodness‐of‐fit and visual predictive checks to assess the level of agreement between observed and model‐predicted concentrations, and the univariate impact of model covariates on model parameters was illustrated (against reference values) in forest plots. Secondary PK parameters for the second dosing interval (including C_trough, Week52_) were derived for the Phase I studies by assuming that a second dose was administered at Day 182.

Similar methodology was employed to develop a PK‐PD model of blood eosinophil count over time, starting with a linear placebo model with an indirect‐response treatment effect. Individual predicted depemokimab PK concentrations, using a dense grid, were used as the exposure measure in the PK‐PD model. Forest plots were used to illustrate covariate effects, as well as treatment effects for a typical participant. Model development and parameter estimations, including estimates of C_max_, *t*_max_, and C_trough_, were performed using NONMEM v7.5 statistical software, while simulations were performed with mrgsolve v1.06 from the R v4.2.2 statistical package.^15^, ^16^

## RESULTS

### PK Analysis

#### PK Analysis Population

In total, 961 participants from the three Phase I and four Phase III studies were included in the PK analysis population. Of these, 529 participants received depemokimab for asthma (36 from the FTIH Asthma study and 493 from SWIFT‐1/‐2) and 272 participants received depemokimab for CRSwNP (from ANCHOR‐1/‐2). A limited number of observations (179 [1.68%]) were below the LLOQ. Baseline characteristics for the PK analysis population are presented in Table 1.

**Table 1:** Baseline Characteristics for the PK Analysis Population, Overall and Stratified by Data Source (Study)

	Single Depemokimab 100 mg Dosing			Two Doses of Depemokimab 100 mg, Every 26 Weeks		
	FTIH Asthma Study (*N* = 36)	China HV Study (*N* = 20)	US HV Study (*N* = 140)	SWIFT‐1/‐2 (*N* = 493)	ANCHOR‐1/‐2 (*N* = 272)	Overall (*N* = 961)
Age, years [min, max]	43.9 (10.6) [20.0, 63.0]	29.7 (6.4) [20.9, 43.5]	35.5 (8.0) [18.0, 50.0]	53.7 (14.8) [12.0, 82.0]	52.4 (13.3) [20.0, 93.0]	49.8 (15.0) [12.0, 93.0]
Body weight, kg	82.7 (10.2)	63.7 (7.21)	72.6 (11.0)	79.2 (19.7)	79.0 (16.0)	78.0 (17.4)
[min, max]	[64.6, 107.0]	[50.9, 79.5]	[51.3, 101.0]	[34.6, 161.0]	[45.9, 145.0]	[34.6, 161.0]
Blood eosinophil count, cells/µL^a^	361 (151)	132 (94.4)	144 (135)	431 (350)	453 (358)	387 (339)
Albumin, g/L^b^	44.2 (2.36)	46.4 (1.69)	45.2 (3.02)	45.4 (3.05)	45.8 (3.13)	45.4 (3.04)
Asian race, n (%)	1 (3)	20 (100)	7 (5)	90 (18)	64 (24)	182 (19)
eGFR, mL/min/1.73m^2^	100 (12.0)	103 (10.9)	104 (17.6)	89.5 (19.1)	92.2 (15.3)	93.0 (18.2)
Injection site						
Upper arm	36 (100)	20 (100)	47 (34)	468 (95)	256 (94)	827 (86)
Thigh	0	0	47 (34)	25 (5)	14 (5)	86 (9)
Abdomen	0	0	46 (33)	0	0	46 (5)
Missing	0	0	0	0	2 (1)	2 (<1)
Study population, n (%)					0	
HV	0	20 (100)	140 (100)	0	0	160 (17)
Asthma	36 (100)	0	0	493 (100)	272 (100)	529 (55)
CRSwNP	0	0	0	0	0	272 (28)
Anti‐drug antibody status, n (%)						
Positive at any point	9 (25)	0 (0)	3 (2)	45 (9)	21 (8)	78 (8)
Negative	27 (75)	20 (100)	137 (98)	447 (91)	251 (92)	882 (92)
Missing	0	0 (0)	0 (0)	1 (<1)	0 (0)	1 (<1)
Maintenance OCS at baseline, n (%)	0	0	0	21 (4)	0	21 (2)

All data are presented as mean (SD) unless otherwise stated.

CRSwNP, chronic rhinosinusitis with nasal polyps; eGFR, estimated glomerular filtration rate; FTIH, first‐time‐in‐human; HV, healthy volunteer; OCS, oral corticosteroid; PK, pharmacokinetic; SD, standard deviation; US, United States.

a The normal range for blood eosinophil count is generally considered to be 0‐500 cells/µL,^29^ although blood eosinophil count for healthy adults has been reported as 30‐330 cells/µL.^30^

b The normal range for albumin is 35‐50 g/L.^31^

#### Depemokimab PK Profiles and Final PK Model

**Figure 1:**
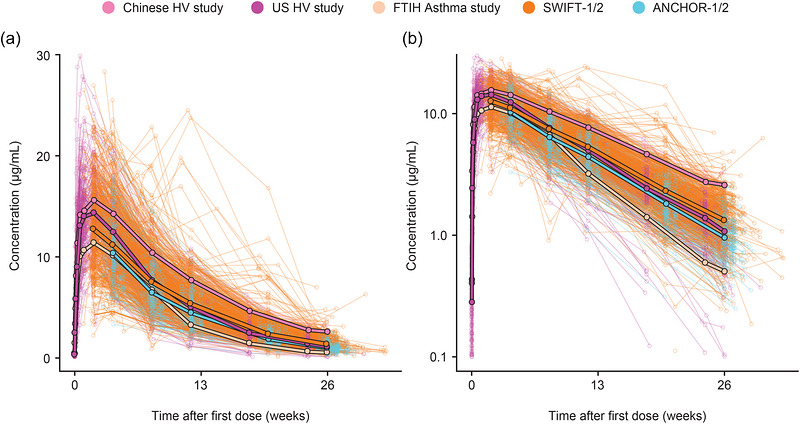
Individual PK profiles following a single 100 mg dose of depemokimab, presented on (a) linear scale and (b) semi‐logarithmic scale (PK analysis population). FTIH, first‐time‐in‐human; HV, healthy volunteer; PK, pharmacokinetic; US, United States.

Overall, individual depemokimab plasma concentration‐versus‐time profiles demonstrated a mono‐exponential decline across all studies (consistent with a one‐compartment PK model), with absorption over approximately 2 weeks followed by slow elimination (Figure 1).

Following structural/base PK model development, stepwise covariate model building and model finalization, the final depemokimab PK model consisted of a one‐compartment model with first‐order absorption and first‐order elimination from the central compartment (Figure S1). The covariates associated with the final model parameters (relevant to the Phase III studies) were: (i) baseline body weight, albumin, estimated glomerular filtration rate (eGFR) and participant population (CRSwNP) on clearance (CL); (ii) baseline body weight and Asian race on volume of distribution (V); (iii) abdomen injection site and baseline age on the first‐order absorption rate constant (*k*_a_); and participant population (CRSwNP) on relative bioavailability (*F*_rel_).

The parameter estimates of the final depemokimab PK model are presented in Table S3, and the individually predicted primary and secondary PK parameters for depemokimab 100 mg are presented in Table 2. Overall, the variability in these PK parameters was low‐to‐moderate between the seven clinical studies, with only minor differences observed between the SWIFT‐1/‐2 (asthma) and ANCHOR‐1/‐2 (CRSwNP) study populations. Specifically, the geometric mean CL/F was 0.093 and 0.100 L/day for the SWIFT‐1/‐2 and ANCHOR‐1/‐2 populations, respectively, and combined with geometric mean V/F values of 6.53 and 6.59 L, this resulted in half‐life values of 48.6 and 45.8 days. In addition, negligible accumulation of depemokimab 100 mg was observed (dosing every 26 weeks), with geometric mean plasma trough levels (C_trough_) consistent at Week 26 and Week 52 for both the SWIFT‐1/‐2 and ANCHOR‐1/‐2 study populations (SWIFT‐1/‐2: 1.23 and 1.32 µg/mL; ANCHOR‐1/‐2: 0.98 and 1.04 µg/mL, respectively).

**Table 2:** Individual Predicted Primary and Secondary PK Parameters Using the Final Depemokimab PK Model (100 mg Dose), Overall and for Each Study

	FTIH Asthma Study (*N* = 9)	China HV Study (*N* = 10)	US HV Study (*N* = 140)	SWIFT‐1/‐2 (*N* = 494)^a^	ANCHOR‐1/‐2 (*N* = 272)	Overall (*N* = 925)
**CL/F, L/day**	0.113 (7.71)	0.063 (12.1)	0.091 (17.4)	0.093 (22.4)	0.100 (19.5)	0.095 (21.5)
**V/F, L**	6.44 (8.78)	5.63 (11.2)	5.76 (13.4)	6.53 (20.7)	6.59 (17.3)	6.41 (19.2)
***k*_a_, /day**	0.38 (27.8)	0.60 (27.8)	0.41 (47.6)	0.20 (25.6)	0.20 (26.0)	0.23 (41.8)
***F*_rel_**	0.88 (9.58)	1.05 (13.8)	0.97 (16.6)	1.01 (14.7)	0.95 (15.9)	0.98 (15.5)
**AUC*_t_*_, ss_, µg·day/mL**	778.8 (8.146)	1661 (12.21)	1071 (23.32)	1081 (27.75)	950.3 (26.57)	1041 (27.76)
***C*_max26‐52_, µg/mL**	12.29 (11.40)	19.50 (14.41)	15.62 (22.38)	13.63 (27.69)	12.46 (27.32)	13.59 (27.88)
**T_max26‐52_, day**	8.41 (19.92)	6.56 (22.44)	8.05 (33.80)	13.59 (17.80)	13.42 (18.00)	12.35 (29.54)
***C*_trough,Week26_, µg/mL**	0.585 (13.38)	2.463 (16.22)	0.987 (43.18)	1.227 (37.00)	0.982 (35.74)	1.11 (40.60)
***C*_trough,Week52_, µg/mL**	0.606 (13.97)	2.779 (17.69)	1.041 (44.79)	1.315 (38.37)	1.041 (37.03)	1.186 (42.28)
***t*_1/2_, day**	39.4 (5.2)	62.0 (9.1)	44.0 (11.8)	48.6 (9.7)	45.8 (9.5)	47.0 (11.1)

All values are geometric mean (%CV).

AUC_*t*, ss_, area under the concentration‐time curve during a dosing interval at steady state; CL/F, apparent clearance; *C*_max26‐52_, maximum concentration during the second dosing interval (Week 26‐52); *C*_trough, Week26_, trough concentration at the end of the first dosing interval (Week 26); *C*_trough, Week52_, trough concentration at the end of the second dosing interval (Week 52); CV, coefficient of variation; *F*_rel_, relative bioavailability; FTIH, first‐time‐in‐human; HV, healthy volunteer; *k*_a_, first‐order absorption rate constant; PK, pharmacokinetic; *t*_1/2_, half‐life; T_max26‐52_, time to maximum concentration during the second dosing interval (Week 26‐52); US, United States; V/F, apparent volume of distribution.

a One participant received a depemokimab dose but did not provide a PK sample; as such they were excluded from the PK analysis dataset but included in the predictive datasets based on their covariates.

**Figure 2:**
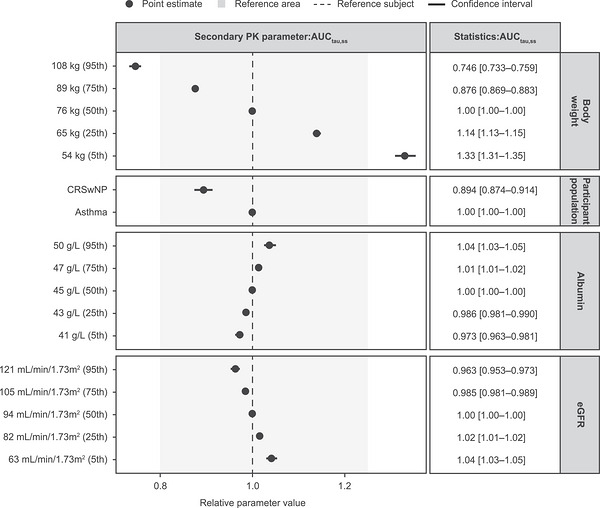
Univariate effects of covariates on depemokimab exposure (AUC_*t*, ss_; final depemokimab PK model). Closed dots represent the median of the predicted relative change from the reference subject and error bars represent 90% CI values. The parameter values for a reference subject (76 kg, 50 years, non‐Asian asthma participant, with baseline albumin of 45 g/L and baseline eGFR of 94 mL/min/1.73 m^2^, receiving a dose of depemokimab 100 mg, into the upper arm) are shown by the dotted vertical line; the shaded area indicates the 80%−125% margins relative to the reference subject and are based on standard bioequivalence limits. Covariates that were tested but not significant (i.e. age, Asian race and injection site) have not been included in this figure. AUC_tau, ss_, area under the concentration‐time curve during a dosing interval at steady state; CI, confidence interval; CRSwNP, chronic rhinosinusitis with nasal polyps; eGFR, estimated glomerular filtration rate; PK, pharmacokinetic.

Visual predictive checks showed good agreement between observed and model‐predicted depemokimab concentrations over time for all study populations (Figure S2). Using the final model, the univariate effect of covariates (relevant to the Phase III asthma and CRSwNP populations) on depemokimab exposure (area under the curve [AUC] at steady state during the dosing interval) are presented in Figure 2, and their effects on trough depemokimab concentrations are presented in Figure S3. Of all the identified covariates, body weight was the major determinant of depemokimab exposure, and satisfied conventional allometry with estimated coefficients of 0.841 for CL/F and 0.887 for V/F, typical for a monoclonal antibody such as depemokimab.^17^ Over the body weight range of 54 to 108 kg (corresponding to the 5th to 95th percentiles), the differences in exposure metrics were limited and not considered clinically relevant. The effects of albumin and eGFR on CL/F were small and therefore not considered clinically relevant (Figure 2). Neither age, race, nor injection site had any meaningful impact on depemokimab exposure. Similar results were seen for depemokimab trough concentrations, although race also appeared to influence C_trough,Week26_ (Figure S3).

### PK/PD Analysis

#### PK/PD Analysis Population

In total, 1324 participants from the FTIH Asthma study and four Phase III studies were included in the PK/PD analysis population. Of these, 796 received depemokimab or placebo for asthma (48 from the FTIH Asthma study and 748 from SWIFT‐1/‐2) and 528 received depemokimab or placebo for CRSwNP (ANCHOR‐1/‐2). Baseline characteristics for the PK/PD analysis population are presented in Table 3. Of note, baseline blood eosinophil counts were higher in the Phase III studies than the Phase I study.

**Table 3:** Baseline Characteristics for the PK/PD Analysis Population, Overall and Stratified by Data Source (Study)

	FTIH asthma study (*N* = 48)	SWIFT‐1/‐2 (*N* = 748)	ANCHOR‐1/‐2 (*N* = 528)	Overall (*N* = 1324)
Age, years [min, max]	44.0 (11.2) [19.0, 65.0]	53.2 (15.1) [12.0, 82.0]	52.0 (13.3) [19.0, 93.0]	52.4 (14.4) [12.0, 93.0]
Body weight, kg [min, max]	82.9 (11.6) [57.8, 107.0]	79.4 (19.7) [34.6, 161.0]	79.4 (16.8) [40.0, 149.0]	79.5 (18.4) [34.6, 161.0]
Blood eosinophil count, cells/µL	366 (147)	433 (374)	456 (551)	440 (449)
Asian race, n (%)	2 (4)	133 (18)	120 (23)	255 (19)
Study population, n (%)				
Asthma	48 (100)	748 (100)	0	796 (60)
CRSwNP	0	0	528 (100)	528 (40)
Regular maintenance OCS at baseline, n (%)	0	40 (5)	0	40 (3)

All data are presented as mean (SD) unless otherwise stated.

CRSwNP, chronic rhinosinusitis with nasal polyps; FTIH, first‐time‐in‐human; OCS, oral corticosteroid; PK/PD, pharmacokinetic/pharmacodynamic; SD, standard deviation.

#### Blood Eosinophil Profiles Following Depemokimab Administration

**Figure 3:**
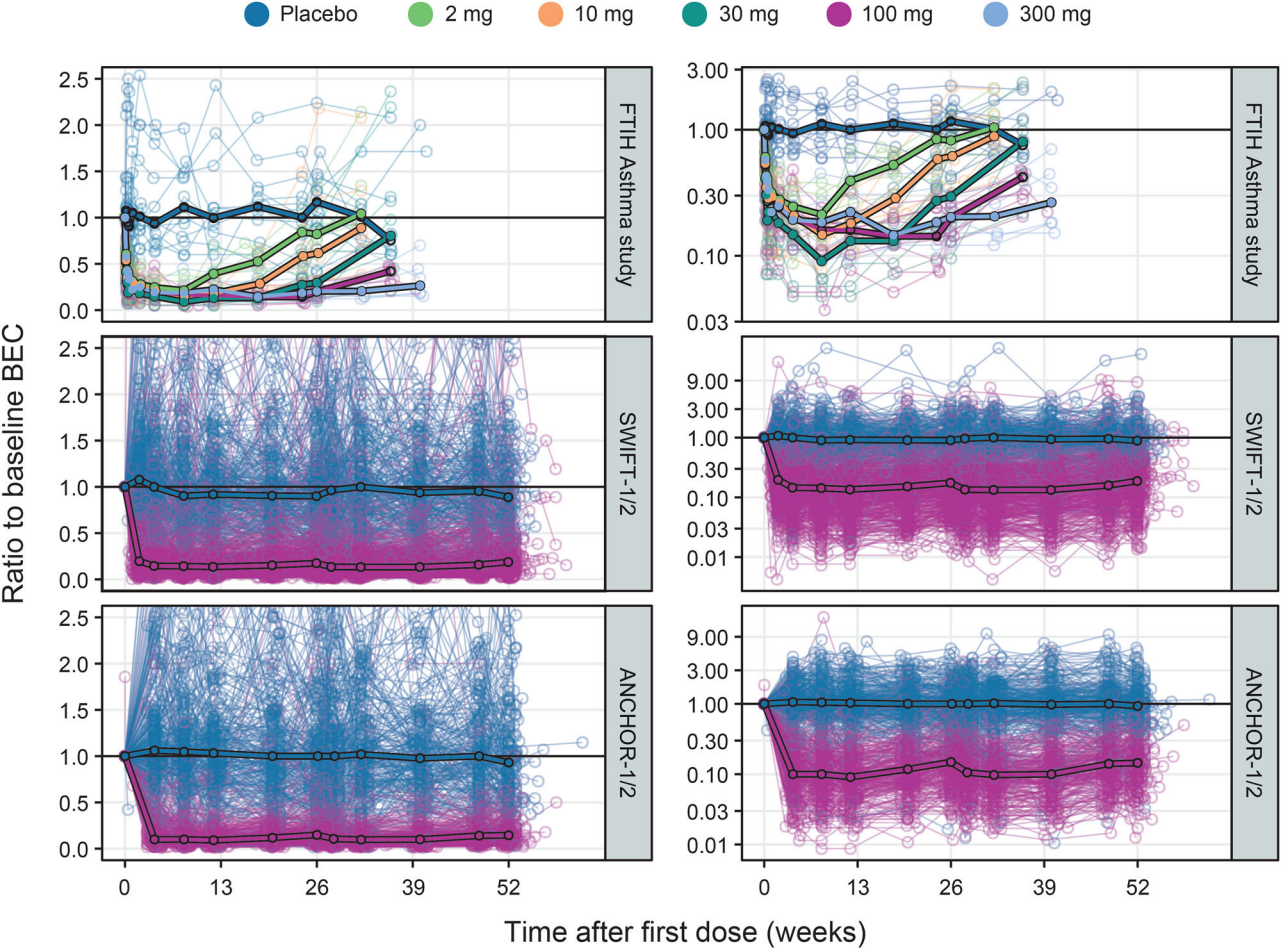
Change from baseline (ratio) in individual blood eosinophil count following depemokimab 100 mg dosing (every 26 weeks). BEC, blood eosinophil count; FTIH, first‐time‐in‐human. Data are presented on linear scale (left) and semi‐logarithmic scale (right).

Blood eosinophil profiles showed that depemokimab rapidly reduced blood eosinophil counts (within 24 hours) across all dosing levels in the FTIH Asthma study with an apparent dose‐dependent duration of effect, while blood eosinophil levels with placebo remained largely unaffected and stable across all studies (Figure 3). Blood eosinophil profiles for the Phase III studies (depemokimab 100 mg) showed a small (<5%) increase in blood eosinophil count prior to the second dose at Week 26 (Figure 3).

#### Final Depemokimab PK/PD Model

The PK/PD model was an indirect‐response model for the treatment effect (blood eosinophil count), including a placebo linear model with baseline and slope for the placebo effect, representing the proportional change over a year during treatment with placebo, plus a numerical solution (differential equation) for the depemokimab treatment effect (Figure S4). Model parameters included baseline blood eosinophil count, depemokimab half‐life on/offset, depemokimab concentration at half maximal effective concentration (EC_50_), maximum effect (E_max_) and the Hill coefficient. The covariates (relevant to the Phase III studies) associated with E_max_ were age, body weight, baseline blood eosinophil count, Asian race and CRSwNP population. The parameter estimates for the final blood eosinophil count model are presented in Table S4. Overall, the depemokimab EC_50_ for blood eosinophil reduction was 0.194 µg/mL and the E_max_ was 84.8%.

**Figure 4:**
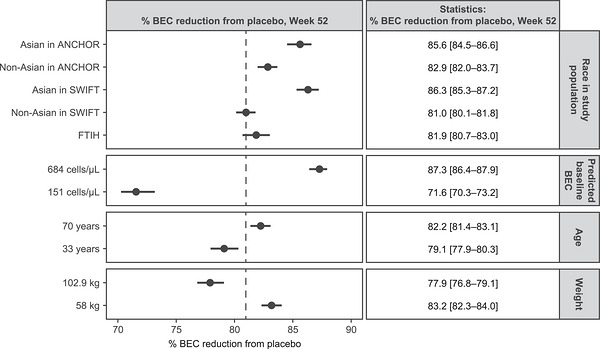
Univariate effects of covariates on adjusted blood eosinophil count (% change vs. placebo) after 52 weeks depemokimab treatment (100 mg every 26 weeks). The covariate effects were based on the PK and PK‐PD models and the predictions were conditioned on a typical reference subject (non‐Asian SWIFT asthma participant population, predicted blood eosinophil count of 317 cells/µL at baseline, 54 years of age, 77.4 kg) shown by the vertical dotted line. The covariate values on the y‐axis were used to generate the parameter predictions and represent the data either as the values of the categorical covariates or as the 10th and 90th percentiles of the continuous covariates. Closed dots represent the median of the predicted (relative) change from the reference subject. Error bars represent 90% CI values associated with the medians. BEC, blood eosinophil count; CI, confidence interval; FTIH, first‐time‐in‐human; PD, pharmacodynamic; PK, pharmacokinetic.

Visual predictive checks showed good agreement between observed and model‐predicted blood eosinophil counts over time for both asthma (FTIH Asthma, SWIFT‐1/‐2) and CRSwNP (ANCHOR‐1/‐2) populations, and showed a sustained reduction over the 26‐week depemokimab dosing intervals (Figure S5). The effect of covariates relevant to the Phase III SWIFT‐1/‐2 and ANCHOR‐1/‐2 populations, (i) blood eosinophil counts relative to placebo (% change) and (ii) absolute blood eosinophil counts, are presented in Figure 4 and S6, respectively. Only baseline blood eosinophil count had a noteworthy impact on placebo‐corrected blood eosinophil count ratio‐to‐baseline at Week 52. Slightly higher responses in the percentage blood eosinophil count reduction from placebo at Week 52 were observed in Asian patients versus non‐Asian patients, older versus younger patients, and patients who had a lower body weight versus higher body weight (<10% difference in the percentage‐point reduction between groups). Across all subgroups, the predicted blood eosinophil count was reduced to 80 cells/µL or lower, and reductions from baseline compared with placebo were maintained close to 80% at Week 52, demonstrating that the magnitude of the covariate effects identified were minor and not of clinical importance.

**Figure 5:**
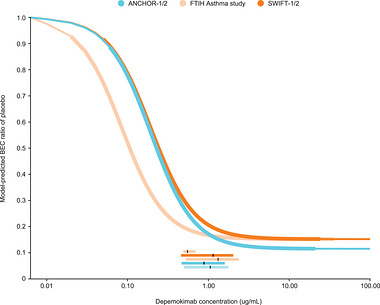
Model‐predicted drug treatment response as a placebo‐corrected blood eosinophil count ratio to baseline. Model‐predicted depemokimab treatment response as blood eosinophil count ratio to baseline ratio to placebo (double ratio) for a theoretical case where depemokimab concentration is held constant, based on the final blood eosinophil count model; figure depicts typical participants belonging to different study populations. The span of the thicker line represents the range of model‐predicted depemokimab concentrations for individual participant populations. The colored bands and the vertical black line represent respectively the predicted 2.5th, 97.5th percentiles and median depemokimab trough concentration at Week 26 (dark colors) and Week 52 (lighter colors) after 100 mg dose administration. Note that participants who did not receive a second dose were not included in the computation of the light colored bands for Week 52. BEC, blood eosinophil count; CRSwNP, chronic rhinosinusitis with nasal polyps; FTIH, first‐time‐in‐human.

The final depemokimab PK model was used to predict individual depemokimab C_trough_ values at Week 52 and assessed against the derived depemokimab potency measures (EC_50_ and concentrations required to obtain 80%, 90%, and 95% of the E_max_ [EC_80_, EC_90_, and EC_95_, respectively]). At Week 52, all participants in the Phase III asthma and CRSwNP populations had a C_trough_ above the EC_50_, and the large majority had C_trough_ values above the EC_90_ (SWIFT‐1/‐2: 94%; ANCHOR‐1/‐2: 83%; combined populations: 90%), suggesting sustained blood eosinophil count suppression at the end of the second dosing interval, as reflected in the model‐predicted exposure‐response for suppression of blood eosinophils (shown in Figure 5).

## Discussion

Overall, the results of this population PK/PD modeling analysis showed that depemokimab was associated with rapid reductions in blood eosinophil count, which were maintained over 52 weeks (based on the SWIFT‐1/‐2 and ANCHOR‐1/‐2 studies) in participants with asthma or CRSwNP. Such reductions in blood eosinophil count suggest sustained suppression of IL‐5 activity (a key driver of type 2 inflammation). This sustained reduction achieves the maximum possible with IL‐5 inhibition, as has been shown with the depemokimab and mepolizumab dose‐response studies, preserving an IL‐5 independent blood eosinophil population, with this residual population characteristically similar to that seen in healthy individuals,^18^, ^19^ and is comparable to the maximal blood eosinophil count reductions observed with mepolizumab in asthma and CRSwNP (and in other type 2‐driven disease states).^11^, ^20^, ^21^, ^22^, ^23^, ^24^ Potency estimates further supported this, with all participants included in the analysis (from randomized, Phase III studies) showing depemokimab C_trough_ levels above the EC_50_ at the end of the dosing interval (with 90% of participants even remaining above the EC_90_ level). Therefore, the results obtained in the current analysis reinforce the PD findings and sustained pharmacology, validating the 100 mg twice‐yearly dosing regimen.

During PK and PD model development, no covariates were identified that resulted in clinically relevant effects on either depemokimab exposure or the PD response on blood eosinophil counts. Body weight is known to influence the PK of monoclonal antibodies.^25^ While weight was impactful on exposure, with upper and lower percentiles falling slightly outside the standard bioequivalence limits (80% to 125% margin), the effect was ultimately not considered to be clinically relevant as it did not appear to affect blood eosinophil count suppression at C_trough_. In addition, findings from the PK/PD model demonstrated the predicted percentage reduction in blood eosinophil count was significant and consistent in magnitude at Week 52 in both the high and low body weight groups. These findings indicate that weight‐based dosing of depemokimab is not required.

In addition, the observed effects of age, race, albumin and eGFR on PK were small and fell within the standard bioequivalence range. The identification of small, non‐clinically relevant effects of these covariates on PK may be due to the extensive PK sampling that was undertaken as part of the Phase III studies; it is possible that with more conventional PK sampling at trough as typically performed in Phase III studies, these would not have been detected. When comparing the China HV and FTIH Asthma studies directly, differences in covariate characteristics besides ethnicity/race should be noted, such as differences in bodyweight, age, albumin, healthy versus disease and bioanalytical assay. The pooled PK dataset included a total of 182 Asian participants, with only 20 participants from the China HV study and 1 from the FTIH Asthma study contributing to the total pool; the remaining participants were from the SWIFT‐1/‐2 and ANCHOR‐1/‐2 studies. As noted above, the observed variation in characteristics between the China HV and FTIH Asthma studies may reflect differences between the studies that were not explicitly tested in the analysis (e.g. differences in bioanalytical assay). For reference, PK parameters from Asian participants in the SWIFT‐1/‐2 and ANCHOR‐1/‐2 studies aligned with the overall Phase III population.

Higher rates of absorption were observed in the HV studies than in the Phase III studies, which may be linked to patients being younger and leaner, but potentially also due to a more consistent SC injection technique owing to the single site nature of the studies and the higher likelihood of the same staff performing all injections. It should also be noted that the absorption rate in these studies versus the Phase III studies was better informed via PK sampling in the absorption phase during the first week. There was also a trend for faster absorption during the first day after abdominal administration (compared with arm or thigh), which may be explained by regional differences in local SC lymph flow and FcRn expression.^26^ Importantly, owing to the long‐acting nature of depemokimab, the impact of variability in absorption rate on relevant parameters such as T_max_, *C*_max_, and AUC was negligible and as such was deemed not clinically relevant. This finding supports previous observations in the US HV relative bioavailability study,^13^ and demonstrates that a flexible approach can be taken with respect to injection site based on the requirements and/or preferences of the participant.

The results of these PK/PD analyses are broadly consistent with those previously reported for the anti‐IL‐5 asthma/CRSwNP treatment mepolizumab, reinforcing the utility of depemokimab as an effective therapy for these conditions. The relationship between mepolizumab PK and blood eosinophil reductions was also described by an indirect‐response model, with mepolizumab also inducing appreciable depletion of blood eosinophil count at higher doses (to <25% of baseline).^27^ Importantly, however, our findings show that depemokimab exhibits extended PK and PD activity compared with mepolizumab.^27^

As with all studies there are limitations that should be considered when interpreting the results. While total IL‐5 levels were sampled in the FTIH Asthma study and Phase III studies, these were not considered relevant for dose selection.^9^ Together with the lack of tissue/bone marrow eosinophil and eosinophil activation marker measurements across all studies included in the analysis, this may have implications for understanding the physiological effects of depemokimab. It should be noted that blood eosinophil count is not a direct surrogate for tissue eosinophil activity (and does not provide an indication of eosinophil activation status), and IL‐5 receptor‐alpha is differentially regulated in blood and airway tissue.^28^ As such, no insights into the dose‐response relationship for depemokimab on tissue eosinophils (or non‐eosinophilic IL‐5 effects in tissue) were gained from this analysis, and future studies and analyses may investigate this to further characterize the efficacy of depemokimab. Moreover, evaluations of the impact of seasonal exacerbation of asthma or chronic rhinitis and concomitant steroid administration may also be of interest.

## Conclusion

In conclusion, results from this PK/PD modeling analysis show that a single dose of depemokimab 100 mg provides durable PD activity throughout the dosing interval, as measured by reduced blood eosinophil counts, suggesting sustained suppression of IL‐5 activity (which is closely linked to type 2 inflammation). In addition, none of the covariates investigated were found to have a clinically relevant impact on either depemokimab exposure or PD activity, indicating no dose adjustments are necessary for either intrinsic or extrinsic factors. Overall, these data support the twice‐yearly recommended depemokimab dosing regimen of 100 mg for patients with asthma or CRSwNP.

## Author Contributions

**AT**, **NB**, and **SS** were involved in study concept or design, data analysis, and data interpretation. **PG** and **IP** were involved in study concept or design, data acquisition and data interpretation. **RF**, **LJ**, and **AG** were involved in study concept or design and data interpretation. **LT**, **AL‐M**, and **JR** were involved in data analysis and data interpretation. **PG** was involved in data interpretation. All authors contributed to the interpretation of the data, the drafting of the manuscript, or its critical revision for important intellectual content, had full access to all data across the studies, and had final responsibility to submit for publication.

## Conflicts of Interest

**AT, RF, LJ, NB, AG, PH,** and **SS** are employees of GSK and hold financial equities in GSK. **LT, ALM,** and **JR** are employees of Pharmetheus AB, which received funding from GSK to conduct this analysis; **JR** also holds financial equities in Pharmetheus AB. **PG** is a paid consultant for AstraZeneca, Eli Lilly, Genentech, GSK, Insmed, Novartis, Regeneron, Roche, and Sanofi. **IP** has received speaker's honoraria for speaking at sponsored meetings from AstraZeneca, Boehringer Ingelheim, Aerocrine, Almirall, Novartis, Teva, Chiesi, Sanofi/Regeneron, Menarini and GSK, and payments for organizing educational events from AstraZeneca, GSK, Sanofi/Regeneron, and Teva; received honoraria for attending advisory panels with Genentech, Sanofi/Regeneron, AstraZeneca, Boehringer Ingelheim, GSK, Novartis, Teva, Merck, Circassia, Chiesi and Knopp, and payments to support FDA approval meetings from GSK; received sponsorship to attend international scientific meetings from Boehringer Ingelheim, GSK, AstraZeneca, Teva and Chiesi.

## Funding

This analysis and the parent studies were funded by GSK (GSK ID: 205722/208021/214099/206713/213744/217095/218079; ClinicalTrials.gov registration: NCT03287310/NCT05140200/NCT05602025/NCT04719832/NCT04718103/NCT05274750/NCT05281523). The sponsor was involved in study design and implementation, as well as data collection, analysis, interpretation, writing the study report and reviewing this manuscript. The sponsor did not place any restrictions on access to data or statements made in the manuscript. All authors had full access to the data upon request and had final responsibility for the decision to submit for publication.

## Supporting information



Supporting information

## Data Availability

Please refer to GSK weblink to access GSK's data sharing policies and as applicable seek anonymized subject level data via the link https://www.gsk‐studyregister.com/en/.
