# Supplementary material for: Population Pharmacokinetics and Pharmacodynamics of Depemokimab in People with Asthma and Chronic Rhinosinusitis with Nasal Polyps
Source: J Clin Pharmacol. 2026 Jul 30;66(8):e70187. doi: 10.1002/jcph.70187 (PMC13422010; doi:10.1002/jcph.70187)
Supplement: Supplementary file 1 — Supporting information [file JCPH-66-0-s001.docx]

## **Supporting information**

**Title:**

Population pharmacokinetics and pharmacodynamics of depemokimab in people with asthma and chronic rhinosinusitis with nasal polyps

**Authors:**

Anders Thorsted, Lénaïg Tanneau, Alexandra Lavalley-Morelle, Richard Follows, Loretta Jacques, Nicholas Bird, Philippe Gevaert, Ian Pavord, Jakob Ribbing, Anubha Gupta, Peter Howarth, Stein Schalkwijk

**Supplementary methods**

Covariate model building

Covariates were categorised into three groups: mechanistic covariates (covariates known to have an impact on one or more parameters of the model), structural covariates (covariates for which there was a strong rationale for expecting an impact on one or more model parameters), and exploratory covariates (covariates that are not mechanistic or structural and that were explored for hypothesis-generating reasons). Mechanistic covariates were included in the base model without statistical testing. Structural covariates were then investigated conditionally on the mechanistic covariates (i.e. the starting point for evaluating the structural covariate-parameter relationships was the base model). The impact of the exploratory covariates was subsequently investigated conditionally on the mechanistic and the identified structural covariates (i.e., the base model that included the identified structural covariate-parameter relationships was the starting point for the evaluation of the exploratory covariate-parameter relationships).

The stepwise covariate model building procedure (SCM) procedure with adaptive scope reduction (ASR) was used for the evaluation of structural and exploratory covariate-parameter relationships. The forward selection and backward elimination p-values were, respectively, 0.01 and 0.001. The ASR threshold p-value was the same as the forward p-value, i.e., 0.01. To further improve the SCM efficiency, the default termination criteria in NONMEM were replaced by CTYPE=4. This restricted the termination check to only consider only the objective function value. In addition, the maximum number of function evaluations allowed (MAXEVAL in NONMEM) was set to 15 times the number of function evaluations required by the base and structural models for the PK model and to 3 and 16 times for the structural and the exploratory SCM for the PK/PD model. Once the model was established according to the above procedure, the default termination criteria were restored and the model was re-run to make sure that it converged successfully. The covariate-parameter relationships evaluated for the PK and PK/PD models are shown in **Supplementary Table S1** and **Supplementary Table S2**.

Non-ordered categorical covariates with more than two categories were binarised into ‘dummy covariates’. For each possible category of these covariates, an additional binary variable was created and assigned a yes/no value. Continuous covariate-parameter relationships were implemented as exponential models, while categorical covariate-parameter relationships were implemented as a fractional difference to the most common category. For the mechanistic covariate body weight, the power relation was used.

The total effect of covariates on a parameter P was then calculated as the product of the n covariate terms in Equation 1:


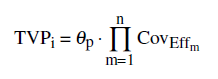


where TVPi is the typical value of the parameter P for subject i and qp is the population typical parameter value (for a subject with typical/reference covariate values).

However, for logit-transformed parameters (maximum effect [E_max_]) covariate-parameter relationships were implemented as additive and, accordingly, the total effect of covariates on such parameter P was then calculated as the addition of the n covariate terms on the logit scale (Equation 2).


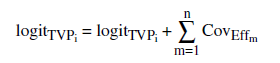


where TVP_i_ is the typical value of the parameter P for subject i and qp is the population typical parameter value (for a subject with typical/reference covariate values). Similarly, for parameters with an additive IIV, such as slope for placebo effect (P_slp_), where different directions needed to be allowed, covariate-parameter relationships were also implemented in an additive form.

#### Supplementary Figure S1. Illustration of the final depemokimab population PK model


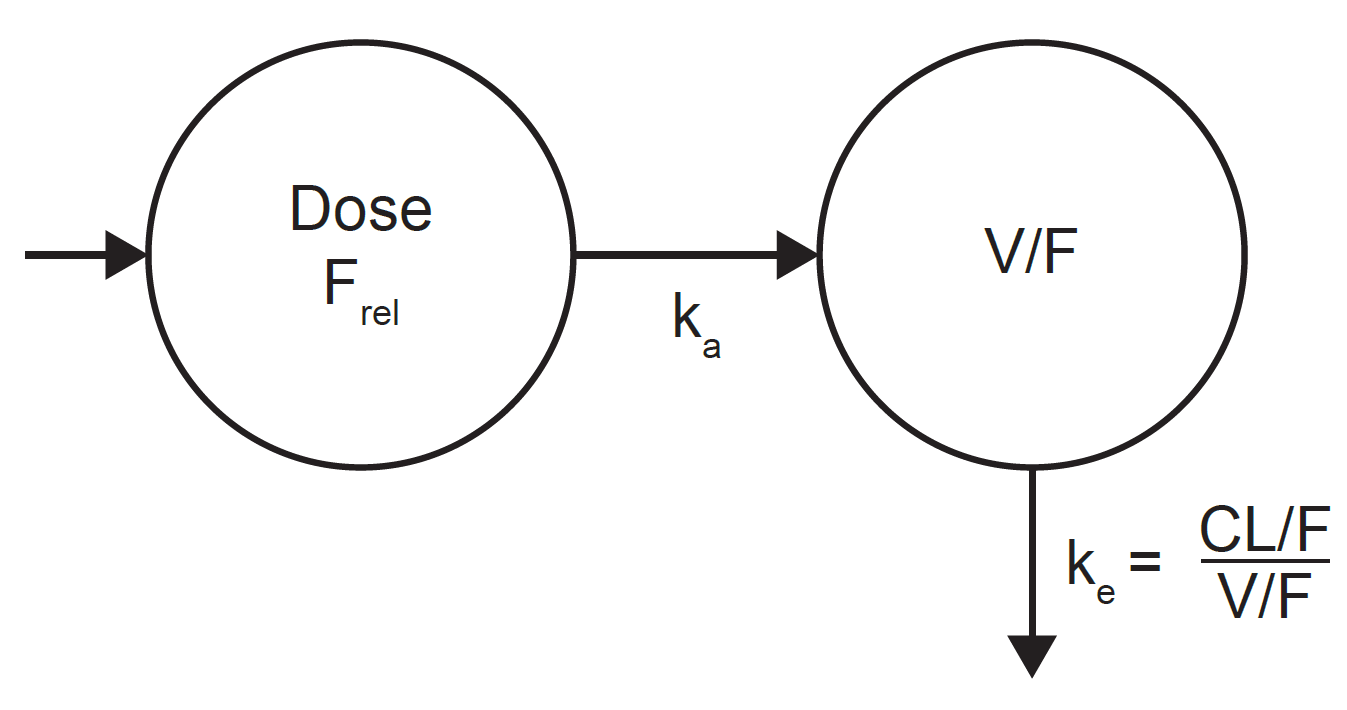


F_rel_, relative bioavailability; CL/F, apparent clearance; k_a_, first-order absorption rate constant; k_e_, first-order elimination rate constant from central compartment; PK, pharmacokinetic; V/F, apparent volume of distribution.

#### Supplementary Figure S2. Visual predictive checks of observed and model-predicted depemokimab (100 mg every 26 weeks) plasma concentrations over time


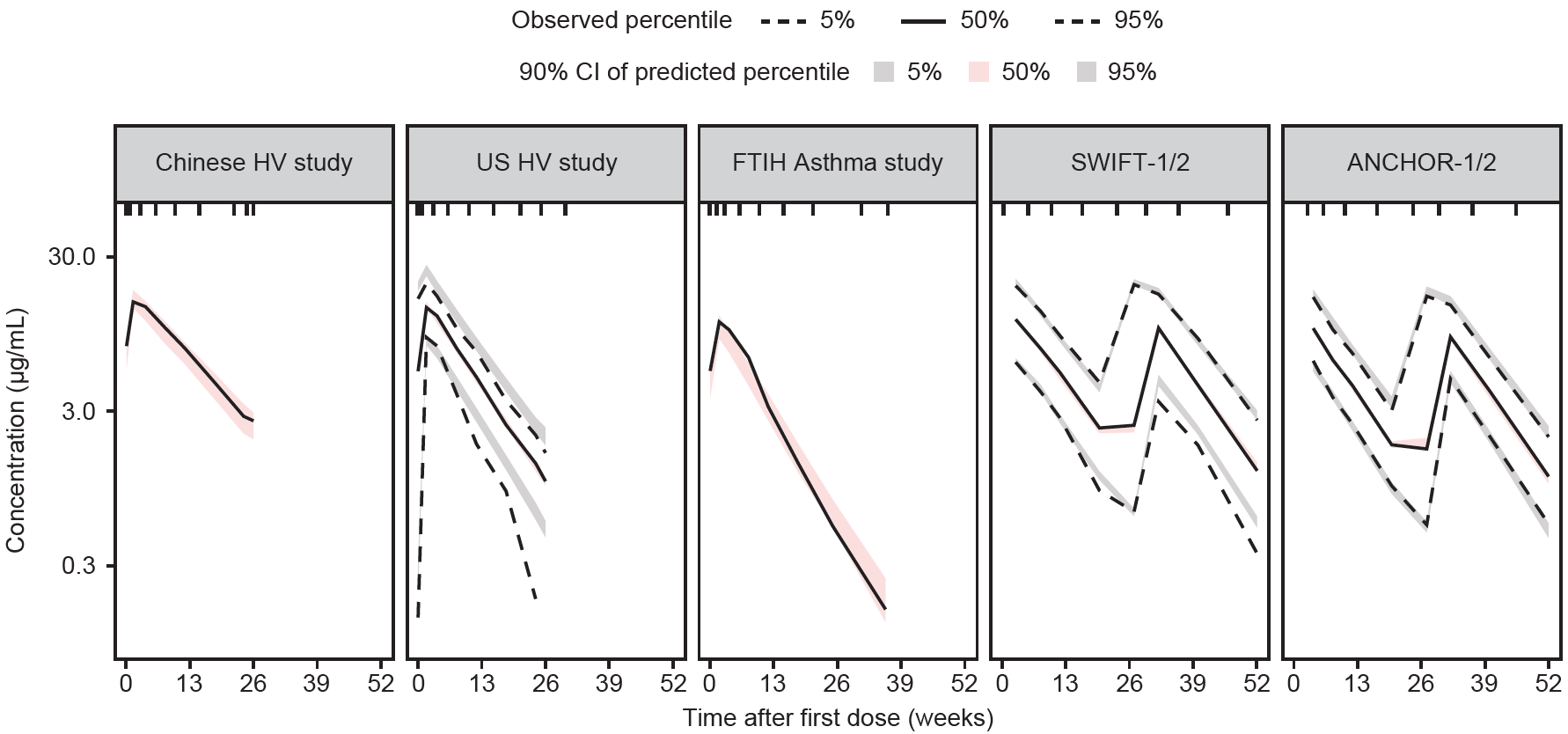


Data are presented on a semi-logarithmic scale. For strata with less than 50 individuals, only the median with its corresponding CI is displayed (China PK HV: n=10; FTIH Asthma: n=9). Time points associated with BLQ observations were included in the visual predictive checks. The black ticks at the top indicate the bins across time.

BLQ, below the limit of quantification; CI, confidence interval; FTIH, first-time-in-human; HV, healthy volunteer; PK, pharmacokinetic; US, United States.

#### Supplementary Figure S3. Univariate effects of covariates on depemokimab trough levels after one 26-week dosing period (C_trough,Week26_; final depemokimab PK model)


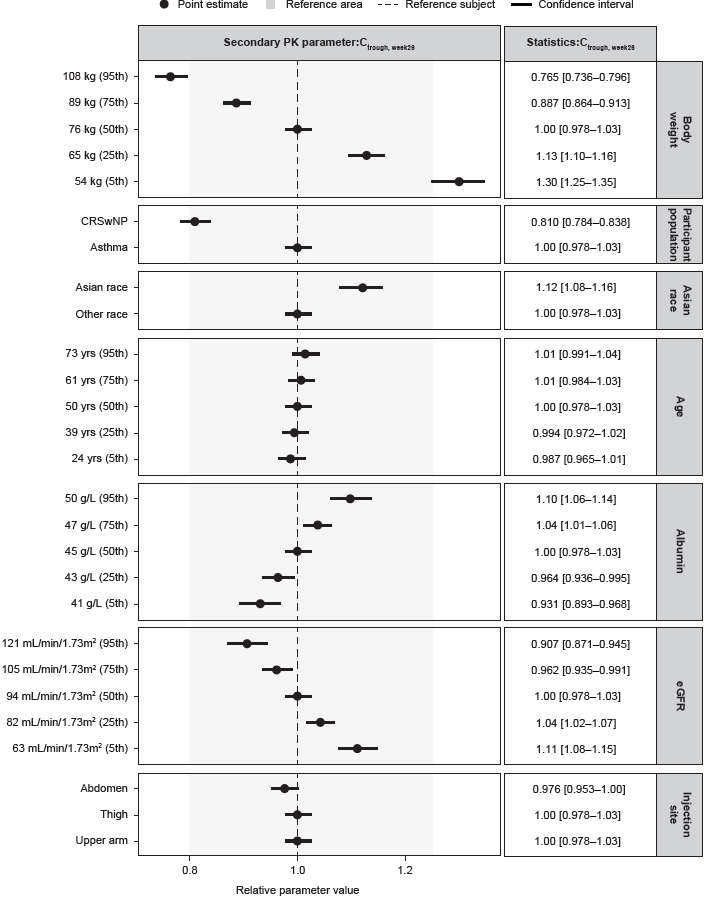


Closed dots represent the median of the predicted relative change from the reference subject and error bars represent 90% CI values. The parameter values for a reference subject (76 kg, 50 years, non-Asian asthma participant, with baseline albumin of 45 g/L and baseline eGFR of 94 mL/min/1.73m², receiving a dose of depemokimab 100 mg, into the upper arm) are shown by the dotted vertical line; the shaded area indicates the 80−125% margins relative to the reference subject and are based on standard bioequivalence limits.

CI, confidence interval; C_trough,Week26_, trough concentration at the end of the first depemokimab dosing interval (Week 26); CRSwNP, chronic rhinosinusitis with nasal polyps; eGFR, estimated glomerular filtration rate; PK, pharmacokinetic.

#### Supplementary Figure S4. Illustration of the final depemokimab blood eosinophil count model


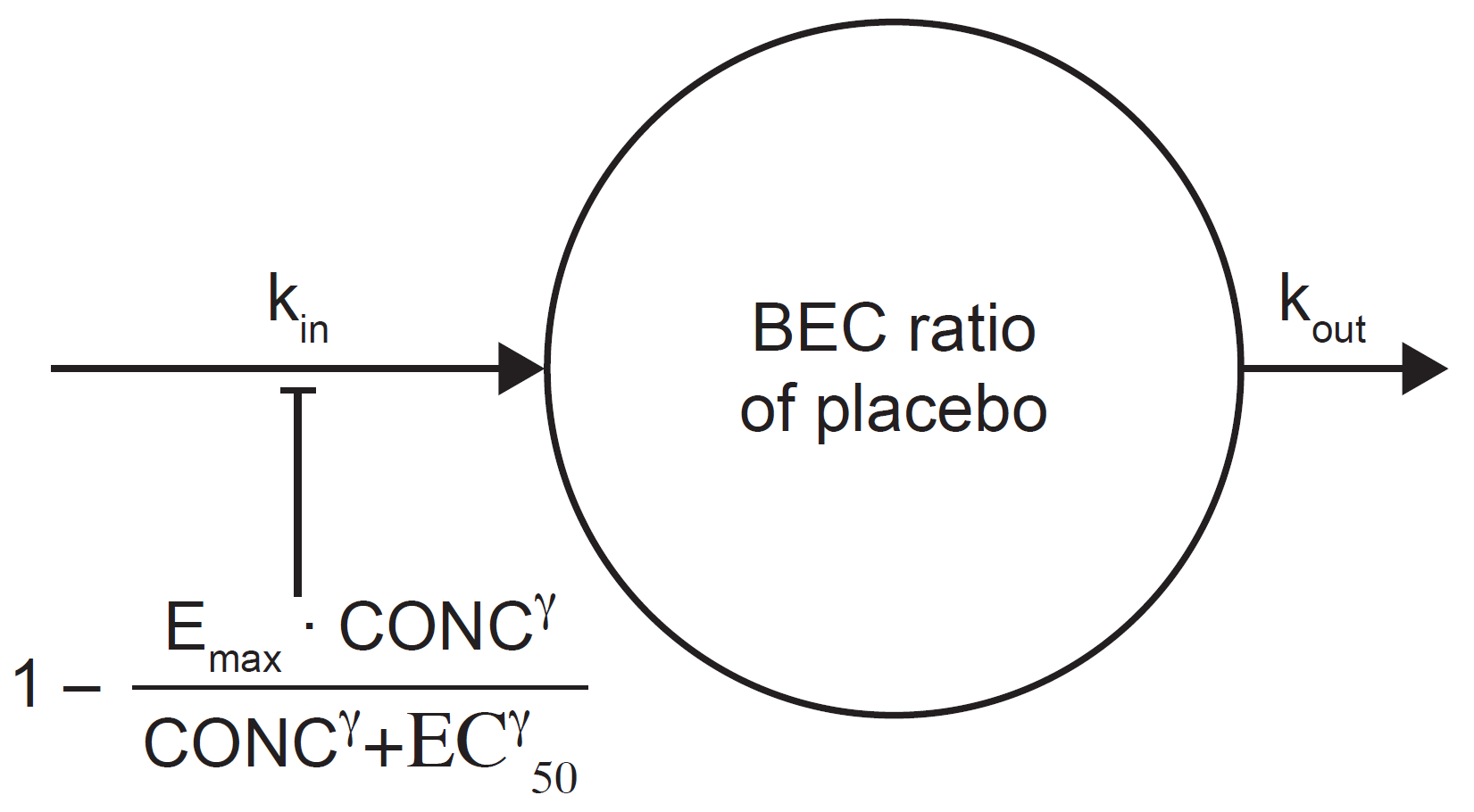


γ, Hill coefficient; BEC, blood eosinophil count; CONC, depemokimab concentration; EC_50_, concentration at half maximum effect; E_max_, maximum effect; k_in_, zero-order production rate constant; k_out_, first-order removal rate constant.

#### Supplementary Figure S5. Visual predictive checks of observed and model-predicted blood eosinophil count over time (change from baseline [ratio]) following one or two doses of depemokimab (100 mg, every 26 weeks)


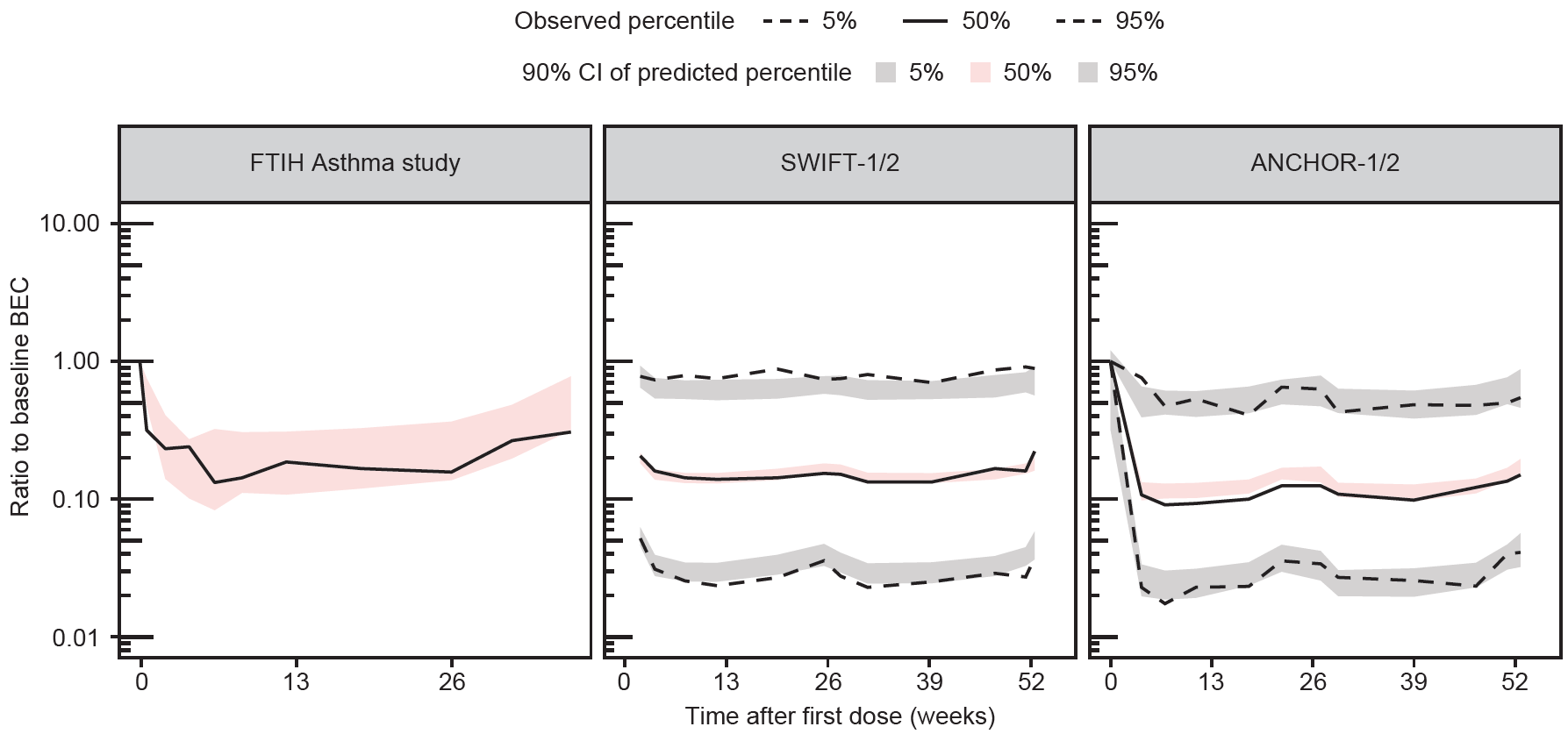


Data are presented on a semi-logarithmic scale. For strata with less than 100 individuals, only the median with its corresponding CI is displayed (FTIH Asthma: n=9). Time points associated with BLQ observations were included in the visual predictive checks. Due to the binning method used (which accounts for the number and distribution of observations), the SWIFT-1/-2 values do not extrapolate back to zero because of differences in sampling times.

BEC, blood eosinophil count; BLQ, below the limit of quantification; CI, confidence interval; FTIH, first-time-in-human.

#### Supplementary Figure S6. Univariate effects of covariates on absolute blood eosinophil count after 52 weeks depemokimab treatment (100 mg every 26 weeks)


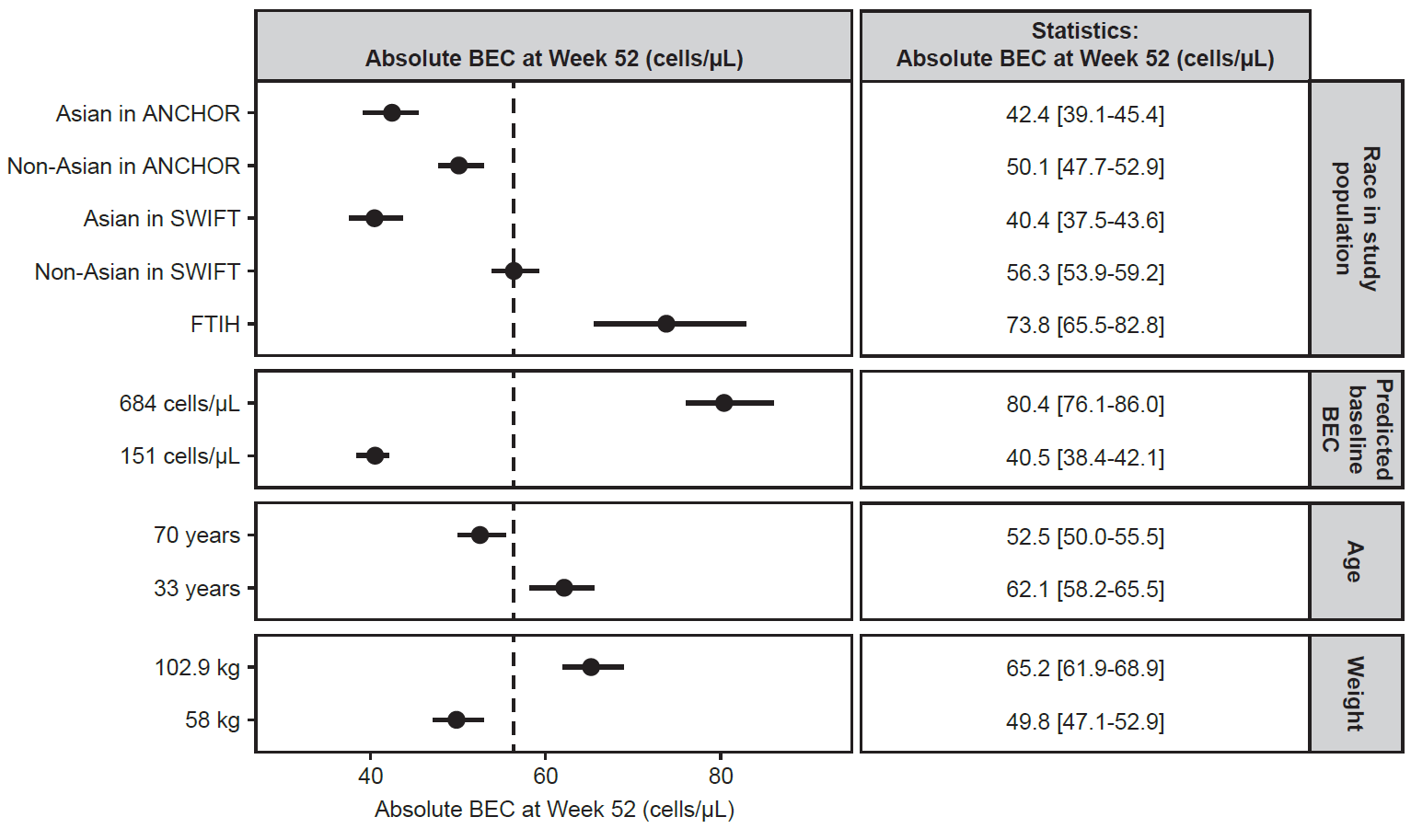


The covariate effects were based on the PK and PK/PD models and the predictions were conditioned on a typical reference subject (non-Asian SWIFT asthma participant population, predicted blood eosinophil count of 317 cells/μL at baseline, 54 years of age, 77.4 kg) shown by the vertical dotted line. The covariate values on the y-axis were used to generate the parameter predictions and represent the data either as the values of the categorical covariates or as the 10th and 90th percentiles of the continuous covariates. Closed dots represent the median of the predicted (relative) change from the reference subject. Error bars represent 90% CI values associated with the medians.

bas., Baseline; BEC, blood eosinophil count; CI, confidence interval; FTIH, first-time-in-human; PD, pharmacodynamic; PK, pharmacokinetic.

#### Supplementary Table S1. Covariate-parameter relationships evaluated in the depemokimab PK model

| **Parameter** | **Type** | **Covariate** |
| --- | --- | --- |
| Clearance | Mechanistic  Structural    Exploratory | Body weight^a^  Albumin, ADA^b^  Age^c^ eGFR^c^, sex, race^d^, baseline eosinophil count, alanine amino transferase, total bilirubin, participant population^e^, study population^e^, 2 mg dose level^f^ |
| Volume of distribution | Mechanistic  Structural    Exploratory | Body weight^a^  N/A  Age sex, race^d^, participant population^e^, study population^e^ |
| First-order absorption rate constant | Mechanistic  Structural      Exploratory | N/A  Body weight^a^, administration device^g^, injection site^g^  Age, sex, race^d^, participant population^e^, study population^e^ |
| Bioavailability | Mechanistic  Structural      Exploratory | N/A  Administration device^g^, injection site^g^  Age, sex, race^d^, participant population^e^, study population^e^, 2 mg dose level^f^ |

^a^Included allometrically, i.e. exponents estimated according to a power model. ^b^Participant’s overall (time-invariant) ADA was tested as a dichotomous covariate. If found significant, after the structural search but before testing exploratory covariates, two alternative ADA covariates could have been tested manually: ADA as time-varying (dichotomous) and ADA as time varying with positive carried forward (so that participant does not go back to negative status after a positive sample). If ADA status would have been found significant in the structural stepwise covariate model building procedure search, ADA titre may also have been tested as a time-varying continuous covariate: similar to ADA as time-varying, ADA titre would then be tested time-varying as observed but also as time varying with cumulative maximum carried forward (so that the peak titre is carried forward in subject with a latter decline in titres). ^c^Age and eGFR were both tested on clearance, even if the absolute correlation was higher than 0.6. ^d^Race categories were driven by the number of participants in each of the seven studies in the analysis dataset. ^e^Participant population and study population together consist of five groups with participant population: I) US HV, II) asthma and III) CRSwNP participants, as well as study differences for IV) China HV and V) FTIH Asthma studies. The remaining three study populations were the reference groups for the three groups in participant population, and therefore not tested for study effects, due to complete confounding with participant population. ^f^Based on higher dose-normalised concentration profile over time compared with other dose groups observed in the FTIH Asthma study. ^g^Only tested for injection sites (thigh or abdomen, vs upper arm) or administration device (autoinjector vs safety syringe device) if the graphical analysis of the US HV study (study 214099) showed a clear trend.

ADA, anti-drug antibody; CRSwNP, chronic rhinosinusitis with nasal polyps; eGFR, estimated glomerular filtration rate; FTIH, first-time-in-human; HV, healthy volunteer; N/A, non-applicable; PK, pharmacokinetic; US, United States.

#### Supplementary Table S2. Covariate-parameter relationships evaluated in the depemokimab PK/PD model

| **Parameter** | **Type** | **Covariate** |
| --- | --- | --- |
| Baseline | Mechanistic  Structural    Exploratory | N/A  Participant population^a^, study population^a^, oral corticosteroid treatment^b^  Age, sex, race/ethnicity^c^, body weight, seasonal month^d^ |
| Placebo response | Mechanistic  Structural    Exploratory | N/A  Baseline eosinophil count^e^  Participant population^a^, study populationa, oral corticosteroid treatment^b^, age, sex, race/ethnicity^c^, body weight |
| Half-life for drug on-/offset^f^ | Mechanistic  Structural    Exploratory | N/A  N/A  Age, body weight, baseline eosinophil count^e^ |
| Maximum effect | Mechanistic  Structural    Exploratory | N/A  Baseline eosinophil count^e^  Participant population^a^, study population^a^, oral corticosteroid treatment^b^, age, sex, race/ethnicity^c^, body weight, NAb^g^ |
| Concentration at half maximum effect | Mechanistic  Structural    Exploratory | N/A  Baseline eosinophil count^e^  Participant population^a^, study population^a^, oral corticosteroid treatment^b^, age, sex, race/ethnicity^c^, body weight, NAb^g^ |

^a^Participant population and study population together consists of three groups with participant population: I) asthma and II) CRSwNP participants, as well as study differences for III) FTIH Asthma study. The HVs were not included in the PK/PD analysis. ^b^Information about oral corticosteroid treatment was tested as a time invariant yes/no in SCM. If found significant, then after SCM oral corticosteroid usage could be tested as a continuous covariate (e.g. corticosteroid dose equivalents in mg) or as the time-variant version of these two covariates, depending on the data quality and suitability. ^c^Race/ethnicity categories were driven by the number of participants in each of the seven studies in the analysis dataset and were defined in the data definition table for the corresponding derived datafile. ^d^Hemisphere-corrected month, explored as a time-varying covariate, with an inflection point mid-July, according to:
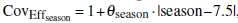
 , where season is a continuous variable ranging between 1 and 13. ^e^If identified by SCM, the observed baseline as covariate may be replaced by the individually-predicted baseline, post SCM. ^f^The covariates to be tested on on-/offset half life are informed by FTIH Asthma study only, since Phase III data did not collect early or washout samples to inform this parameter. ^g^Participant’s overall (time-invariant) NAb status will be tested as a dichotomous covariate in the exploratory SCM search. If found significant, two alternative NAb covariates may be tested manually (in model finalisation): NAb as time-varying (dichotomous) and NAb as time varying with positive carried forward (so that participant does not go back to negative status after a positive sample).

CRSwNP, chronic rhinosinusitis with nasal polyps; FTIH, first-time in human; HV, healthy volunteer; N/A, non-applicable; NAb, neutralising antibody; PK/PD, pharmacokinetic/pharmacodynamic; SCM stepwise covariate model building procedure.

#### Supplementary Table S3. Parameter estimates for the final depemokimab PK model

|  |  |  | **Final PK model** | |
| --- | --- | --- | --- | --- |
| Run |  |  | 11 | |
| OFV |  |  | -15637.4 | |
| Condition number |  |  | 43.13 | |
|  | **Unit** | **Value** | **RSE (%)** | **SHR (%)** |
| CL/F | L/day | 0.0920 | 0.860 |  |
| V/F | L | 6.29 | 0.997 |  |
| WT on CL | allo. exponent | 0.841 | 3.78 |  |
| WT on V | allo. exponent | 0.887 | 3.64 |  |
| F_rel_ |  | 1.00 | [FIX] |  |
| k_a_ | /day | 0.212 | 4.05 |  |
| TAD <3 h on RUV | ^a^ | 5.18 | 7.58 |  |
| CRSwNP participant population on CL | frac. change | 0.0625 | 16.3 |  |
| Albumin on CL | frac. change per g/L | -0.00684 | 23.2 |  |
| eGFR on CL | frac. change per mL/min/1.73m^2^ | 0.00134 | 17.1 |  |
| Asian race on V | frac. change | 0.0788 | 17.5 |  |
| CRSwNP participant population on F_rel_ | frac. change | -0.0509 | 29.3 |  |
| Age on k_a_ | frac. change per year of age | -0.00735 | 31.3 |  |
| Abdomen injection site on k_a_ | frac. change | 0.520 | 23.9 |  |
| FTIH Asthma study population on CL | frac. change | 0.155 | 12.9 |  |
| China HV study population on CL | frac. change | -0.219 | 8.79 |  |
| 2 mg dose on CL | frac. change | -0.349 | 71.1 |  |
| HV participant population on V | frac. change | -0.0513 | 24.9 |  |
| FTIH Asthma study population on F_rel_ | frac. change | -0.173 | 17.2 |  |
| HV participant population on k_a_ | frac. change | 0.588 | 21.0 |  |
| FTIH Asthma study population on k_a_ | frac. change | 0.627 | 23.6 |  |
| China HV study population on k_a_ | frac. change | 0.405 | 27.9 |  |
| IIV CL | CV | 0.101 | 6.06 | 10.2 |
| IIV F_rel_ | CV | 0.167 | 3.52 | 8.49 |
| IIV k_a_ | CV | 0.351 | 6.37 | 24.7 |
| Correlation IIV F_rel_ - IIV k_a_ | Cor | 0.638 | 6.74 |  |
| IIV RUV | CV | 0.488 | 4.20 | 0.361 |
| RUV | CV | 0.178 | 1.95 | 2.04 |

^a^Defined as the fractional change in RUV magnitude.

The IIV and RUV parameters are reported on the SD scale, which for exponential IIV and additive RUV on the log-scale corresponds to the approximate CV scale. The RSE for IIV and RUV parameters are reported on the approximate SD scale. The RSE for the correlation is reported for the square root of the eta covariance. The continuous covariate effects are defined as the fractional change in parameter per one unit change in covariate. Allometric exponents are unitless as defined

with power models.

allo., allometric; CL/F, apparent clearance; Cor, correlation; CRSwNP, chronic rhinosinusitis with nasal polyps; CV, coefficient of variation; eGFR, estimated glomerular filtration rate; F_rel_, relative bioavailability; frac., fractional; FTIH, first-time-in-human; HV, healthy volunteer; IIV, inter-individual variability; k_a_, first-order absorption rate constant; OFV, objective function value; PK, pharmacokinetic; RSE, relative standard error; RUV, residual unexplained variability; SD, standard deviation; SHR, shrinkage; TAD, time after dose; V/F, apparent volume of distribution; WT, body weight.

#### Supplementary Table S4. Parameter estimates for the final blood eosinophil count model

|  |  |  | **Final model** | |
| --- | --- | --- | --- | --- |
| Run |  |  | 18 | |
| OFV |  |  | -2683.68 | |
| Condition number |  |  | 38.7 | |
|  | **Unit** | **Final model Value** | **RSE (%)** | **SHR (%)** |
| Baseline | Cells/μL | 317 | 2.05 |  |
| P_slp_^a^ | frac. change per year | -0.0466 | 29.5 |  |
| HL_onset_ | Day | 3.08 | 4.73 |  |
| E_max_^b^ |  | 0.848 | 0.564 |  |
| EC_50_^a^ | μg/mL | 0.194 | 10.3 |  |
| Hill coefficient |  | 1.64 | 6.11 |  |
| Predicted baseline blood eosinophils on E_max_^c^ | add. change on logit scale | 0.834 | 4.93 |  |
| CRSwNP participant population on E_max_ | add. change on logit scale | 0.320 | 14.7 |  |
| Age on E_max_ | add. change on logit scale per year of age | 0.00660 | 21.4 |  |
| Weight on E_max_ | add. change on logit scale per kg | -0.00471 | 24.4 |  |
| Asian race in Asthma population on E_max_ | add. change on logit scale | 0.450 | 15.3 |  |
| Asian race in CRSwNP population on E_max_ | add. change on logit scale | 0.187 | 40.2 |  |
| FTIH Asthma study population on EC_50_ | frac. change | -0.561 | 9.12 |  |
| FTIH Asthma study population on P_slp_ | add. change | 0.357 | 29.8 |  |
| Change in RUV for FTIH Asthma study population | frac. change | -0.242 | 17.9 |  |
| IIV RUV | CV | 0.396 | 2.75 | 7.64 |
| IIV baseline | CV | 0.646 | 2.89 | 6.94 |
| IIV E_max_^d^ | SD | 0.497 | 3.96 | 9.31 |
| RUV^a^ | CV | 0.426 | 1.38 | 1.65 |

^a^These values represent typical estimates for participants included in the Phase III studies.

^b^This value represents the typical estimate for a non-Asian asthma participant, defined as one with a with predicted baseline blood eosinophil count of 317 cells/mL, an age of 54 years and a body weight of 77.4 kg.

^c^This represents the change in logit E_max_ per log unit change in predicted baseline blood eosinophil count centred around the log median.

^d^The IIV is presented as the SD on the logit scale. The corresponding CV for E_max_ was 0.0756, for the typical non-Asian participant from the SWIFT Asthma study. The CV was calculated using the following approximation: SD = θ⋅(1 – q)⋅θ⋅SDlogit and CV = SD/θ.

The RSE for IIV and RUV parameters are reported on the approximate CV scale, with the exception of the IIV for E_max_.

add., additive; CRSwNP, chronic rhinosinusitis with nasal polyps; CV, coefficient of variation; EC_50_, concentration at half maximum effect; E_max_, maximum effect; frac., fractional; FTIH, first-time-in-human; HL_onset_, depemokimab half-life on/offset; IIV, inter-individual variability; OFV, objective function value; P_slp_, slope for placebo effect; RSE, relative standard error; RUV, residual unexplained variability; SD, standard deviation; SHR, shrinkage.
